# Impact of acute partial-body cryostimulation on cognitive performance, cerebral oxygenation, and cardiac autonomic activity

**DOI:** 10.1038/s41598-021-87089-y

**Published:** 2021-04-08

**Authors:** Dimitri Theurot, Benoit Dugué, Wafa Douzi, Paul Guitet, Julien Louis, Olivier Dupuy

**Affiliations:** 1 Laboratory MOVE (EA 6314), Faculty of Sport Sciences, University of Poitiers, 8 allée Jean Monnet, 86000 Poitiers, France; 2 Research Institute for Sport and Exercise Sciences (RISES), Liverpool John Moores University, Byrom Street, Liverpool, L3 3AF UK; 3 Ecole de Kinésiologie et des Sciences de l’Actvivité Physique (EKSAP), Faculté de Medecine, Université de Montreal, Montreal, Canada

**Keywords:** Neurophysiology, Physiology

## Abstract

We assessed the effects of a 3-min partial-body cryostimulation (PBC) exposure—where the whole body is exposed to extreme cold, except the head—on cognitive inhibition performance and the possible implications of parasympathetic cardiac control and cerebral oxygenation. In a randomized controlled counterbalanced cross-over design, eighteen healthy young adults (nine males and nine females) completed a cognitive Stroop task before and after one single session of PBC (3-min exposure at − 150 °C cold air) and a control condition (3 min at room temperature, 20 °C). During the cognitive task, heart rate variability (HRV) and cerebral oxygenation of the prefrontal cortex were measured using heart rate monitoring and near-infrared spectroscopy methods. We also recorded the cerebral oxygenation during the PBC session. Stroop performance after PBC exposure was enhanced (562.0 ± 40.2 ms) compared to pre-PBC (602.0 ± 56.4 ms; *P* < 0.042) in males only, accompanied by an increase (*P* < 0.05) in HRV indices of parasympathetic tone, in greater proportion in males compared to females. During PBC, cerebral oxygenation decreased in a similar proportion in males and females but the cerebral extraction (deoxyhemoglobin: ΔHHb) remained higher after exposure in males, only. These data demonstrate that a single PBC session enhances the cognitive inhibition performance on a Stroop task in males, partly mediated by a greater parasympathetic cardiac control and greater cerebral oxygenation. The effects of PBC on cognitive function seem different in females, possibly explained by a different sensitivity to cold stimulation.

## Introduction

The therapeutic effects of cold have been known for a very long time. Hippocrates (460–370 BC) already recommended the use of local cold application through ice and snow to relieve pain. In contemporary times, winter swimming regularly takes place in the form of bath in ice-cold water in certain countries^1^. Nowadays, local and systemic cold applications using various cryostimulation techniques (e.g. cold water immersion, ice application, partial- and whole-body cryostimulation) are widely used for therapeutic purposes, including the control of inflammation, pain, and swelling associated with certain pathologies^2–4^.

In sports medicine, the use of whole- or partial-body cryostimulation (WBC or PBC, exposition of the whole body or the whole body except the head, respectively, to a very cold air) has gained popularity for its systemic effects on the organism, in particular to accelerate the post-exercise recovery process. For example, WBC and PBC were shown to reduce muscle pain sensations^5,6^, reduce inflammation^7–9^, improve sleep^10,11^, and possibly enhance recovery through an increased activation of the parasympathetic tone of the autonomic nervous system^10,12–15^ and muscle tissue oxygenation^16^. However, despite the plethora of studies conducted in the last decade, the physiological underpinning of PBC and WBC is not well known, and in particular its effects on the central nervous system and most particularly on cognitive function.

Cognitive function refers to mental processes taking part in the acquisition and treatment of external information^17^. These mental processes are various and include functions such as memory, attention, and executive functions^18^. Good cognitive functioning is essential in daily life activities and processes such as executive function, which classically refers to inhibition, working memory, and cognitive flexibility allowing the realization of complex cognitive tasks^19^. Cognitive processes also play a major role in physical performance. Indeed, many sporting disciplines require strong executive functions, such as cognitive flexibility and inhibition, in order to make timely decisions and perform at one’s best.

Executive functions, mainly under the control of the prefrontal cortex, are affected by several physiological mechanisms such as neuroendocrine responses and oxygen availability. Based on the neurovisceral integration model, heart rate variability (HRV) and executive functions are linked through the prefrontal neural function^20,21^, while the vagal related control of the myocardium is related to the prefrontal cortex activity^22^. Previous studies evaluating the link between cardiac parasympathetic activation (measured through HRV indices), and performance of executive functions, revealed that a higher vagal tone was associated with better executive functioning^23,24^. On the contrary, decreased HRV indices of parasympathetic activation and increased sympathetic activation were linked to reduced performances of executive functions^25^. Moreover, increases in HRV indices of cardiac vagal control following aerobic exercise training were associated with enhanced performances of inhibition^26^. Brain oxygen availability also plays a major role in cognitive performance and increased cerebral oxygenation seems to be related to better executive function^27–30^. On the contrary, decreased cerebral oxygen availability as shown in different experimental conditions (i.e., exercise or hypoxia) negatively altered cognitive performance^31^.

The influence of short-term cold exposure on human brain functioning and cognitive functions has not received much attention. Amongst the few studies conducted so far, a decrease in core body temperature following body exposure to cold would have a detrimental effect on cognitive performance^18,32–34^ including executive functions^32^. This detrimental effect would be explained by the distraction theory according to which the cold stimuli may interfere with the focus that should be put to the completion of a given cognitive task^6^. However, most of these studies evaluated cognitive performance during or following long-duration exposures to cold, such as two hours in 10 °C cold air^32^ or after cold water immersion^34,35^. The shorter exposure times and more extreme cold stimuli proposed in WBC and PBC systems expose people to a potential other range of physiological stress, which effects on cognitive function and performance are unknown. To date, only Patil et al.^36^, reported an increase of alertness but a deterioration of short-term memory after a 3-min whole-body immersion in cold water. Furthermore, recent studies assessing the impact of head or face exposure to cold, recorded improvements in cognitive performance in stroop tasks^37,38^.

Therefore, the aim of the present study was to examine the effect of a single 3-min PBC session on executive cognitive performance with a particular focus on the inhibition process. Based on current scientific evidence showing that cryostimulation exposure enhances cardiac autonomic vagal control and tissue oxygenation, we hypothesized that PBC exposure would enhance cognitive inhibition performance through an increased cardiac vagal tone and cerebral oxygenation.

## Materials and method

### Participants

Eighteen healthy participants (nine males and nine females) volunteered to participate in this study (see Table 1 for participants' characteristics). All participants were physically active, from 20 to 30 years old, and were not accustomed to cold exposure or any cryostimulation treatments. Participants were checked for contraindication to cold exposure such as Raynaud syndrome, cold allergy, acute infection, or cardiovascular and circulatory diseases. Besides, inclusion criteria for female participants required that they were all under monophasic oral contraception which resulted in a consistent concentration of sex hormones throughout the different sessions of data collection^39^. Participants were asked not to drink alcohol, coffee, or practice strenuous physical exercise for 24 h prior to each experimental session. All participants were volunteers and received an information leaflet explaining the protocol and measures. They were also informed about the risks and benefits of their participation as well as their rights according to the declaration of Helsinki and signed a written informed consent. This protocol and all the methods and measures used in this study were in accordance with the recommendations and guidelines provided by the declaration of Helsinki. The study was approved by the local Ethics Committee (Comité d’Ethique pour la Recherche en STAPS number: 2019-26-05-34).

**Table 1:** Characteristics of participants.

	Age	Height	Weight	BMI	Fat mass %
Male	22.3 ± 1.8	177.3 ± 4.9	77.7 ± 9.7	24.6 ± 2.5	15.2 ± 5.5
Female	22.8 ± 1.4	162.0 ± 6.0*	57.5 ± 9.4*	21.8 ± 2.3*	25.3 ± 5.0*

Data are presented mean ± SD.

*Significant difference between male and female P < 0.05.

### Experimental design

#### Familiarization trial

The familiarization trial consisted of an inclusion visit during which the participants were checked for contraindication to participation. If all criteria for participation were met, anthropological measures were obtained, and participants undertook a familiarization Stroop task for the cognitive inhibition task. The familiarization trial was set 1 week prior to the experimental trial.

#### Experimental trial

**Figure 1:**
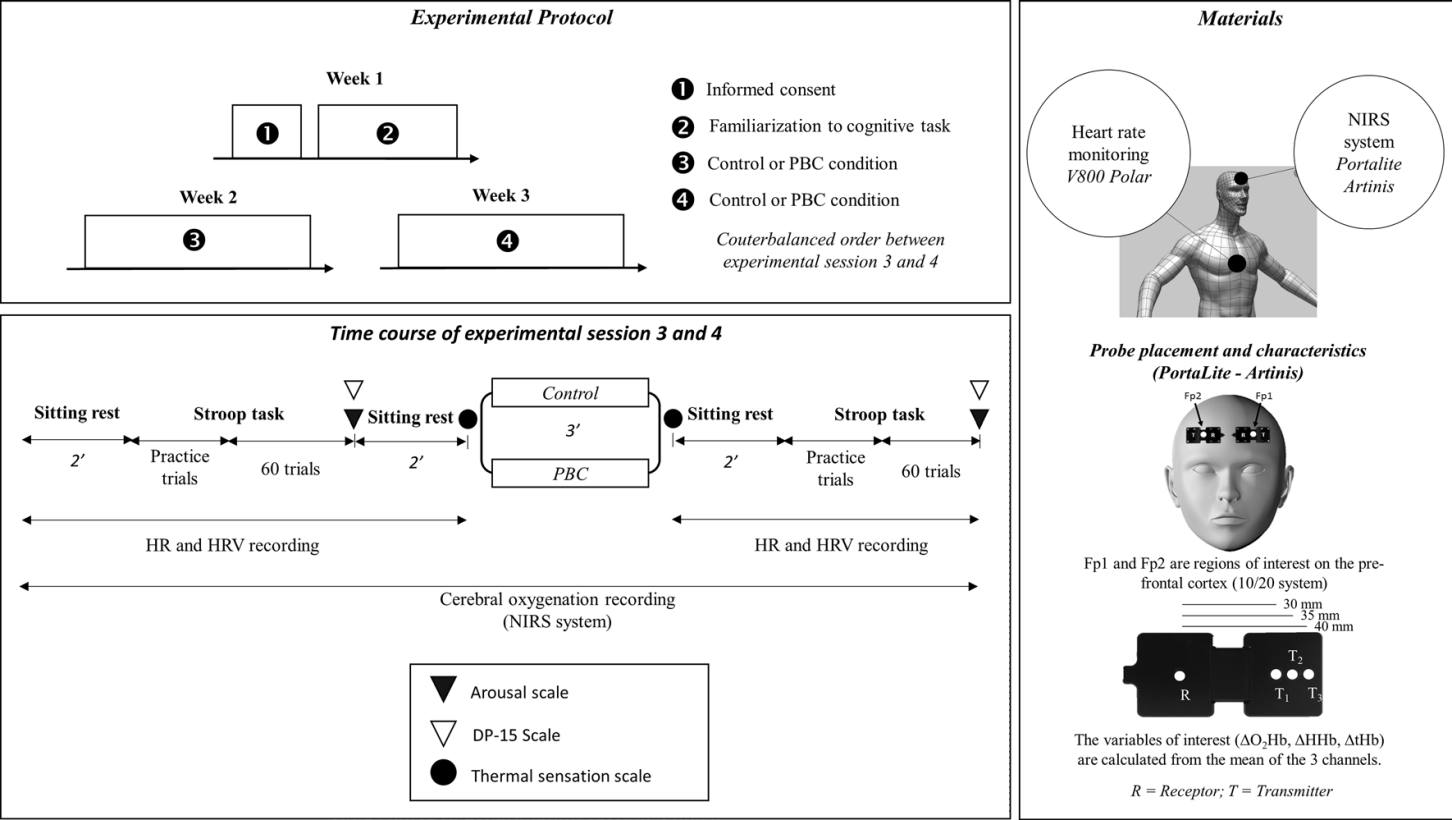
Schematic of the experimental protocol. *PBC* partial body cryotherapy.

The protocol was composed of one testing session per week for 2 consecutive weeks at the same time of the day. Using a coin flip, participants were randomly assigned either to partial-body cryostimulation (PBC at − 150 °C) or control testing condition (ambient room temperature ≈ 20–21 °C) before the first session and changed condition for the second session, so that they were tested in a randomized, counterbalanced, cross-over design. This procedure was performed by the principal investigator and applied for male and female participants separately. Upon arrival at the laboratory, participants were asked to dress in a swimsuit and were given a bathrobe. Following a 5-min resting period during which the participants remained seated, a first Stroop task was performed. Immediately after the Stroop task, participants undertook either a 3-min PBC session or stayed 3 min standing in the cryosauna at ambient room temperature. Following the PBC session or the CONT condition, another 3-min resting period was scheduled, following which the participants performed a second Stroop task. Subjective measurements were assessed upon completion of each Stroop task. During the cognitive tasks and resting periods, the participants wore a heart rate monitor and a Near-Infrared Spectroscopy (NIRS) system to continuously measure HRV, and prefrontal cortex oxygenation, respectively. Figure 1 displays a schematic of the experimental design.

### Experimental measurements

#### Anthropometric measures

One week prior to the experimental session, the participants’ height was measured to the nearest 0.1 cm using a wall stadiometer. Bodyweight and composition were assessed by using a validated^40^ 8-point bio-impedance device (Tanita BC418-MA, Tanita Corp., Tokyo, Japan).

#### Partial-body cryostimulation session

PBC sessions were performed in a cryosauna (CryoJet, ICE MINI, Paris, France) under the supervision of trained personal. The cryosauna was pre-cooled at − 70 °C following what participants were asked to enter the cryosauna only wearing a swimsuit, a pair of gloves, and socks. They were asked to take off all metallic items such as necklaces or rings to avoid any frostbites when exposed to extreme cold. The temperature of the cryosauna was automatically maintained at − 150 °C until the end of the 3-min session. In the CONT condition, the protocol was identical to the PBC condition, except that the cold was not applied. A PBC system exposing the whole body to cold air except the head was utilized in this study instead of a WBC system so that cerebral oxygenation could be recorded continuously during the session.

#### Cerebral oxygenation measurement

Cerebral oxygenation was assessed through the non-invasive Near-Infrared Spectroscopy (NIRS) technique, using two Portalite Systems (Artinis Medical Systems B. V., Elst, The Netherlands). During the protocol, left and right prefrontal cortex oxyhemoglobin (ΔO_2_Hb), deoxyhemoglobin (ΔHHb), and total hemoglobin (ΔtHb) were continuously recorded. Optodes were placed on the forehead upon the Fp1 and Fp2 regions of the international 10–20 system and were maintained with an elastic bandage. This placement probes to measure prefrontal cortex oxygenation, as previously described in several studies^29,30,41^. Sampling frequency was set at 10 Hz. Baseline values for the signal were means of the last 1 min of the resting period before the first Stroop task (before experimental exposition). All values recorded during the Stroop task were normalized according to baseline values and averaged between the left and right NIRS systems. For all NIRS parameters, average values during the two Stroop tasks were calculated for further statistical analyses.

#### HRV measurement

HRV was measured through R–R interval analysis using a heart rate monitor^42^ positioned onto the participants’ chest (V800, Polar Electro Oy, Kempele, Finland) during the entire duration of the protocol, except during PBC sessions. To avoid HR fluctuations, the test was performed in a quiet and dimmed-light room, and participants remained still in a sitting position. Time series of R–R intervals were extracted using the Polar Flow software and analyzed with Kubios software (v. 3.3.1, 2019, Finland). The HRV analysis concentrated on data collected during the Stroop tasks performed before and after the PBC or control session.

R–R intervals during 128 s (to fit with the duration of the Stroop task) were analyzed using time-domain indices (mean HR and root mean square of the successive differences, RMSSD), frequency-domain indices (low-frequency band, LFnu; high-frequency band, HFnu, and LF/HF ratio) and nonlinear indices (standard deviations perpendicular to, SD1 in a Poincaré plot analysis), which were associated to parasympathetic tone.

#### Computerized Stroop test

A modified computerized Stroop task was used to assess cognitive inhibition process. In the current study, the task was composed of four-color words (red, blue, green, and yellow), which were presented on a black screen. The four color words were presented in one of four color inks (red, blue, green, yellow) but always incongruent with the meaning of the word. A trial was constituted of a 500 ms cue (a white cross in the middle of the screen), followed by a color word appearance for 3000 ms in the center of the screen. Participants were asked to indicate the color of the ink by pressing the button corresponding to the right color. The answers were mapped to the letters “e”, “r”, “o”, and “p” on an AZERTY keyboard, which participants used to give their answers with the right and left hand. The mapping remained the same throughout the task. The order was for the left hand: “middle finger—red”, “index finger—green” and for the right hand: “index finger—blue”, and “middle finger—yellow”. Participants performed 15 familiarization trials followed by 60 experimental trials during which the reaction time (ms) and accuracy (% of good responses) were recorded. During practice and experimental trials, a visual feedback (“Error”) was given for incorrect responses only. Participants were asked to answer as fast as possible making as few errors as possible. This procedure was previously used in several studies^28,30,43^. This test is known to assess specifically the inhibition process of executive functions and has been previously used in the validation of the Neurovisceral Integration model, which suggests a functional link between HRV and reaction time.

#### Perceptual measures

Perceptual measures were conducted through visual analogic scales (VAS). At the end of each Stroop task, perceived arousal^44^ was obtained using a five-point VAS ranging from 1 to 5 (1 = low arousal, 5 = high arousal) and perceived task difficulty^45^ was assessed using the 15-point DP-15 VAS ranging from 1 to 15 (1 = extremely easy, 4 = very easy, 6 = easy , 8 = slightly difficult, 10 = difficult, 12 = very difficult, 15 = extremely difficult). In addition, immediately before and after the PBC session or control session, cold sensation was recorded using an eleven-point VAS from 0 to 10 (0 = neutral, 2 = slightly cool, 4 = cool, 6 = cold, 8 = very cold, 10 = unbearable cold) to assess thermal perception^46^.

### Statistical analysis

All data are presented as mean ± standard deviation (SD). A 3-way analysis of variance (Condition [CONT vs. PBC] × Sex [male vs. female] × Time [pre vs. post]) for repeated measures was performed to analyze the effect of PBC, contingent upon sex, on reaction time and accuracy to inhibition during the Stroop test, HRV parameters, perceptual responses and cerebral oxygenation. When a significant interaction between time, condition, and/or sex was found, post-hoc analyses were performed using the Tukey HSD test. A t-student test was performed to compare anthropometric characteristics of males and females. For all statistical analyses, *P* values ≤ 0.05 indicate significance. Effect sizes (EF) were also calculated using Hedge’s formula and interpreted with Cohen’s scale, where EF ≤ 0.2 (trivial), > 0.2 (small), > 0.5 (moderate), > 0.8 (large) and > 1.2 (very large).

## Results

### Anthropometric characteristics

All anthropometric characteristics of participants are presented in Table 1. Sex differences were found for all subject characteristics except for age. Height, body weight, and BMI were higher in males (P < 0.05 for each), and % fat mass (P < 0.05) was lower in males compared to females.

### Perceptual responses

**Figure 2:**
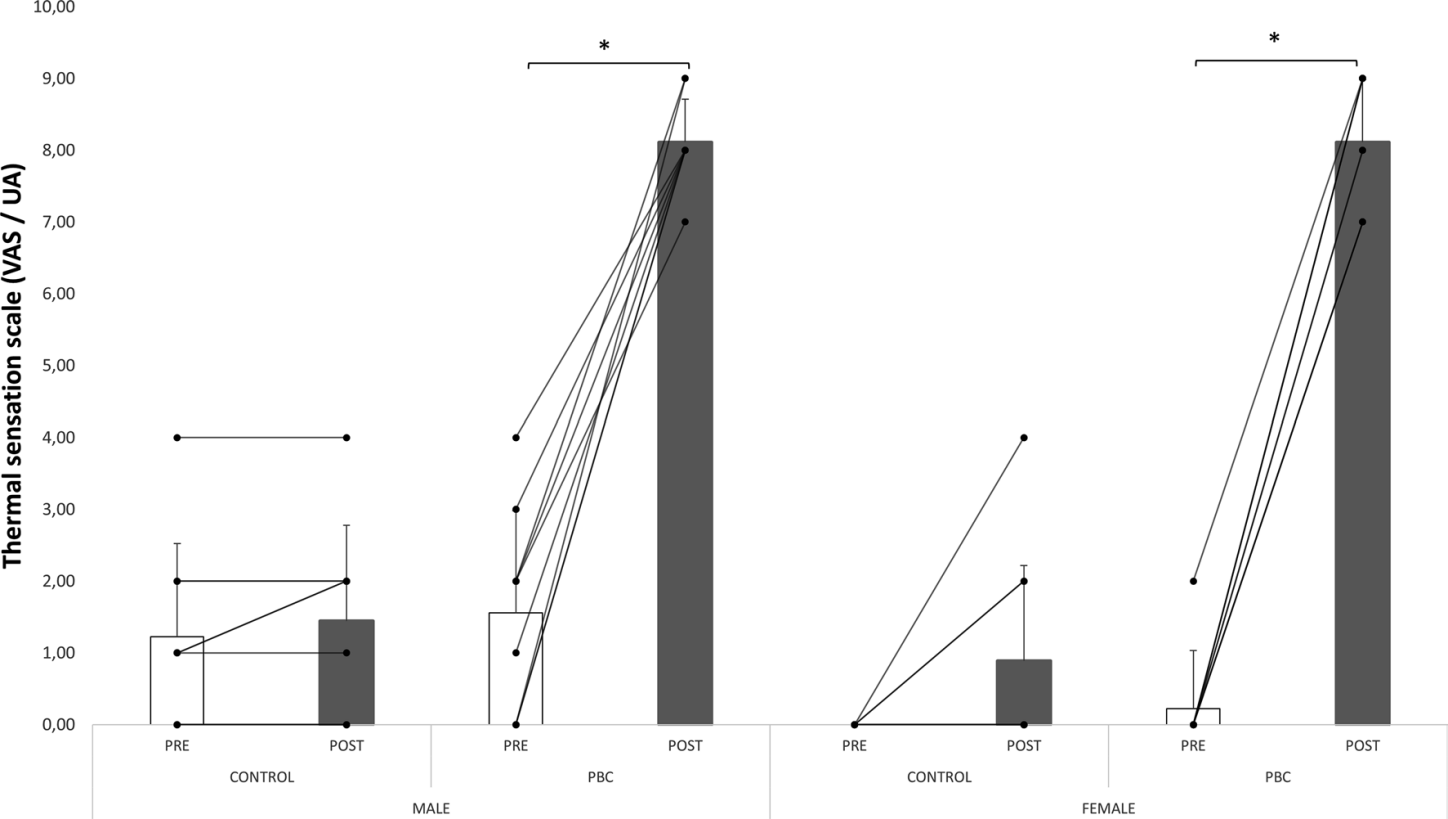
Cold sensation before and after experimental conditions. *PBC* partial body cryotherapy. *Significant (*P* < 0.05).

The ANOVA revealed a significant interaction between condition and time for cold sensation. Following PBC, cold sensation increased in both males and females (Fig. 2). Furthermore, no significant effect of time nor condition was reported for the scores of the arousal scale and DP-15 scale (Table 2).

**Table 2:** Subjective measurements during the inhibition task of the Stroop test.

	Control				PBC			
	Male		Female		Male		Female	
	Pre	Post	Pre	Post	Pre	Post	Pre	Post
DP-15 scale	5.67 ± 1.94	6.00 ± 2.06	6.11 ± 1.54	5.78 ± 1.79	5.22 ± 1.56	5.00 ± 1.94	6.33 ± 2.00	5.22 ± 1.48
Arousal scale	3.67 ± 1.12	3.89 ± 1.27	3.89 ± 0.60	4.33 ± 1.00	3.78 ± 0.97	4.56 ± 1.01	3.78 ± 0.97	4.44 ± 1.01

Data are presented as mean ± SD.

*PBC* partial body cryotherapy.

*Significant p < 0.05.

### Stroop test

**Figure 3:**
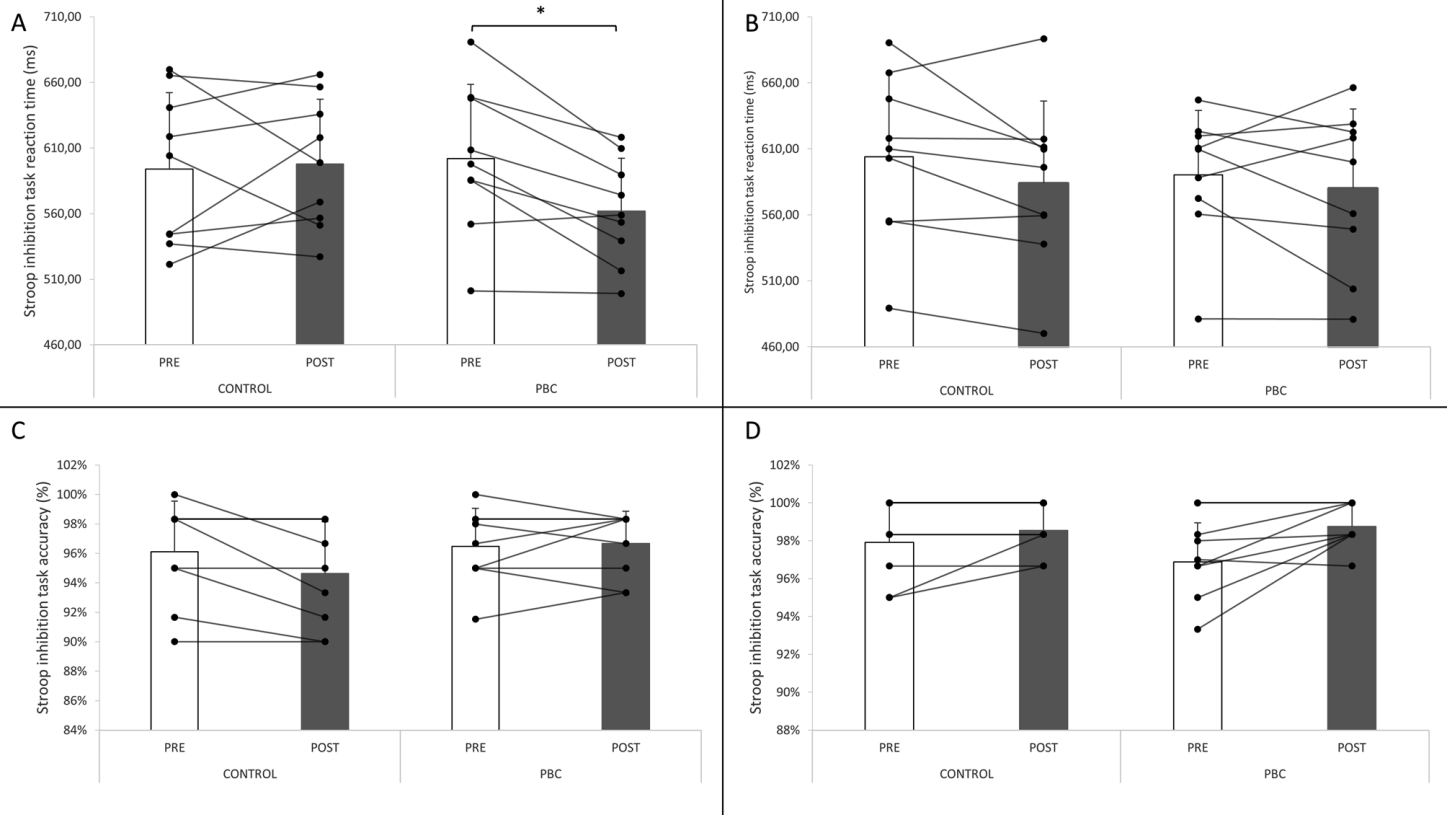
Reaction time and accuracy to the Stroop task in males (**A** and **C**) and females (**B** and **D**) before and after PBC (partial body cryotherapy) and control conditions. *Significant (*P* < 0.05).

Stroop test reaction time and accuracy to the inhibition task are presented in Fig. 3. The ANOVA revealed a significant interaction between time, sex, and condition (*P* = 0.033) for reaction time. Post-hoc analyses revealed a significantly faster reaction time post-PBC (562.0 ± 40.2 ms) compared to pre-PBC (602.0 ± 56.4 ms) only in males (*P* = 0.042). No effect of time or condition was noted for accuracy to the task.

### HRV parameters

**Figure 4:**
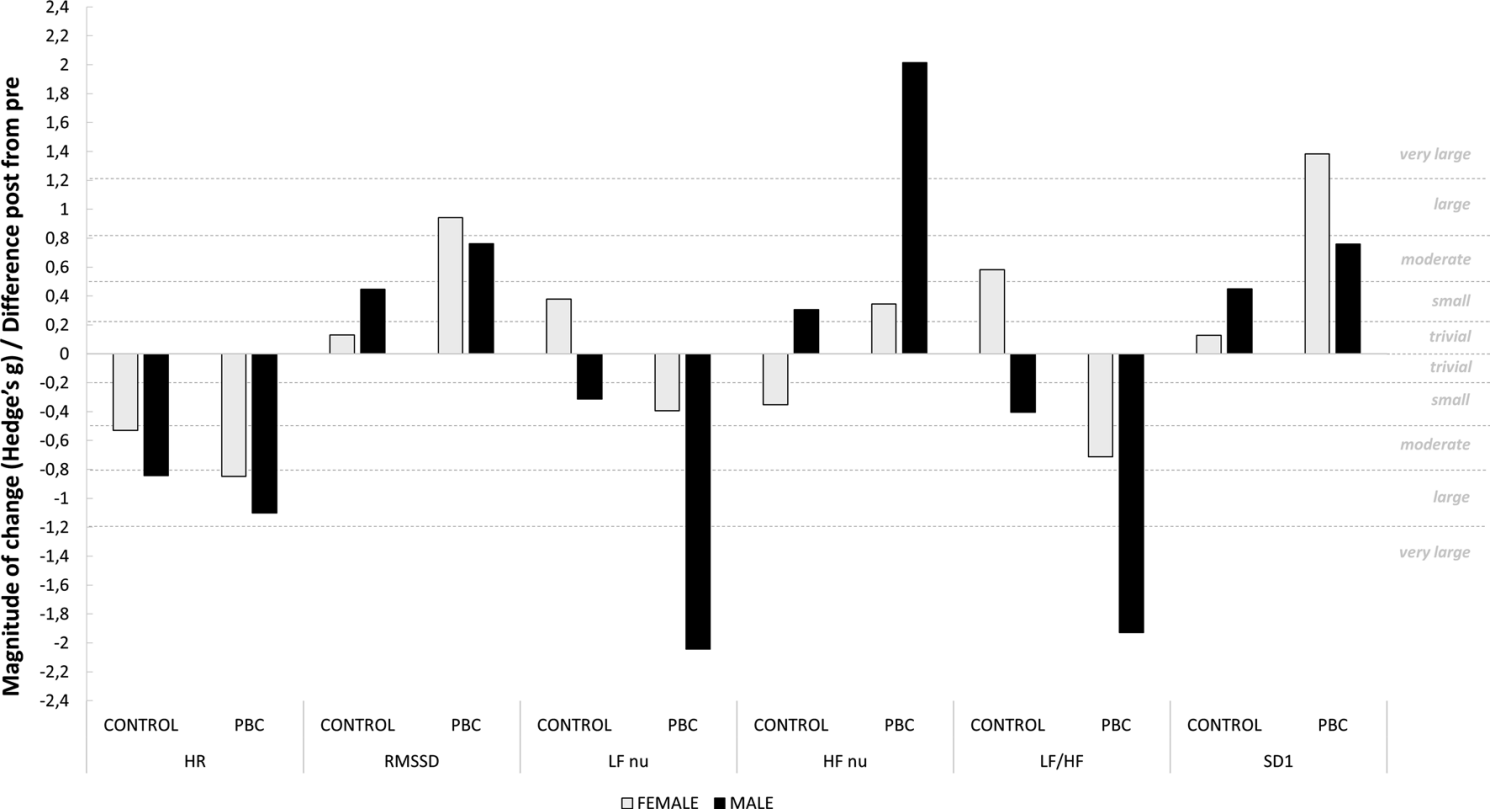
Magnitude of change of heart rate variability indices from prior to PBC (partial body cryotherapy) and control conditions.

Prior to PBC exposure, all HRV parameters were identical between conditions. The evolution of HRV parameters between pre and post PBC session is displayed in Table 3. We also pooled values of males and females as no significant effect of sex was revealed by the ANOVA. However, EF calculations display important differences between males and females (Fig. 4). Pooled data of males and females revealed a significant interaction between time and condition for HR (*P* < 0.01), RMSSD (*P* < 0.05), LFnu (*P* < 0.01), HFnu (*P* < 0.01), LF/HF (*P* < 0.05) and SD1 (*P* < 0.05). Post-hoc analyzes revealed a significant increase in RMSSD (pre-PBC: 30.0 ± 16.3 vs. post-PBC: 56.2 ± 39.4 ms, *P* < 0.01), HFnu (pre-PBC: 33.5 ± 18.6 vs. post-PBC: 54.0 ± 20.3 ms^2^, *P* < 0.001) and SD1 (pre-PBC: 21.3 ± 11.6 vs. post-PBC: 39.9 ± 28.0 ms, *P* < 0.001) following the PBC exposure compared to prior PBC exposure. On the contrary, a significant decrease in HR (pre-PBC: 86.7 ± 12.2 vs. post-PBC: 74.1 ± 12.0 beats min^−1^, *P* < 0.001) and LFnu (Pre-PBC: 66.2 ± 18.7 vs. post-PBC: 44.8 ± 20.9 ms^2^, *P* < 0.001) was observed following the PBC exposure compared to prior PBC exposure. In the Control condition, HR was also significantly lower (*P* < 0.001) during the post-Control (HR = 80.5 ± 9.7 bpm) when compared to pre-Control (HR = 87.2 ± 10.4 bpm). Also, no differences between conditions were found for LF/HF when post-hoc analyzes were conducted.

**Table 3:** Heart rate variabilty responses to partial body cryotherapy.

Inhibition task	Total Group		Male		Female	
	Control	PBC	Control	PBC	Control	PBC
**Mean HR (beats.min**^**-1**^**)**						
PRE	87.2 ± 10.4	86.7 ± 12.2	83.8 ± 8.2	85.6 ± 11.6	90.6 ± 11.7	88.0 ± 13.5
POST	80.5 ± 9.7*	74.1 ± 12.0*	76.6 ± 8.1	72.9 ± 10.3	84.5 ± 9.9	75.6 ± 14.2
**RMSSD (ms)**						
PRE	31.1 ± 16.1	30.0 ± 16.3	36.0 ± 16.5	31.4 ± 19.1	26.2 ± 15.1	28.5 ± 13.7
POST	37.4 ± 22.7	56.2 ± 39.4*	46.6 ± 27.4	60.7 ± 48.2	28.1 ± 12.2	51.1 ± 29.0
**LF nu (ms**^**2**^**)**						
PRE	59.2 ± 21.1	66.2 ± 18.7	59.5 ± 17.0	71.8 ± 11.0	58.9 ± 25.7	59.9 ± 24.0
POST	61.7 ± 23.9	44.8 ± 20.9*	53.8 ± 17.3	40.4 ± 17.6	69.5 ± 27.8	49.8 ± 24.4
**HF nu (ms**^**2**^**)**						
PRE	40.3 ± 21.0	33.5 ± 18.6	40.4 ± 17.0	28.1 ± 10.9	40.2 ± 25.5	39.6 ± 24.0
POST	38.1 ± 23.9	54.0 ± 20.3*	45.9 ± 17.3	59.3 ± 17.8	30.3 ± 27.8	48.1 ± 22.5
**LF/HF**						
PRE	2.5 ± 2.7	3.5 ± 4.1	2.0 ± 1.7	3.0 ± 1.4	3.0 ± 3.4	4.0 ± 5.9
POST	3.6 ± 4.4	1.6 ± 2.9	1.5 ± 0.9	0.8 ± 0.6	5.8 ± 5.5	2.4 ± 4.1
**SD1 (ms)**						
PRE	22.1 ± 11.4	21.3 ± 11.6	25.5 ± 11.7	22.3 ± 13.5	18.6 ± 10.7	20.2 ± 9.7
POST	26.5 ± 16.1	39.9 ± 28.0*	33.1 ± 19.4	43.1 ± 34.3	19.9 ± 8.7	36.3 ± 20.6

Data are presented as mean ± SD.

*PBC* partial body cryotherapy.

*POST significantly different from PRE (P < 0.05).

### Cerebral oxygenation during PBC or control

**Figure 5:**
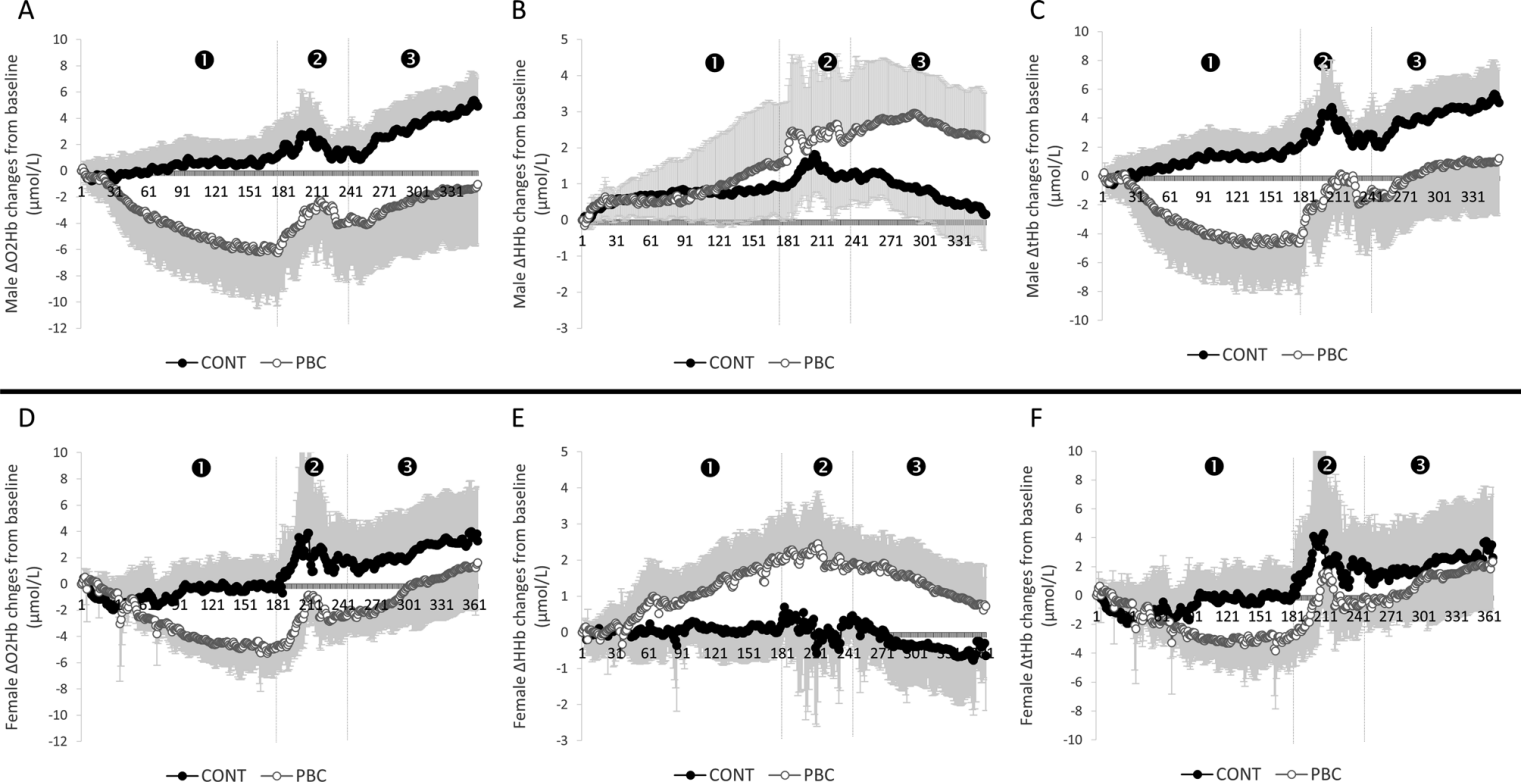
Changes in cerebral oxygenation during and after the 3-min cryostimulation and control (CONT) conditions. 1—PBC/ 2—exit of PBC (1 min)/ 3—sitting time (2 min). (**A**–**C**) Shows the kinetics of ΔO_2_Hb, ΔHHb and ΔtHb, respectively, in males. (**D**–**F**) Show ΔO_2_Hb, ΔHHb, and ΔtHb in females. *PBC* partial body cryotherapy, *O*_*2*_*Hb* oxyhemoglobin, *HHb* deoxyhemoglobin, *tHb* total hemoglobin. Significant differences between conditions (P < 0.05) are presented in text for more clarity.

The cerebral changes during experimental conditions are presented in Fig. 5. The changes of concentrations were expressed from the first second of the exposition. During PBC or control, ΔO_2_Hb and ΔtHb displayed the same pattern. The ANOVA revealed a main effect of time (P < 0.05), and a significant interaction between condition and time (P < 0.05). ΔtHb and ΔO_2_Hb decreased from the 50th s after the beginning of the PBC session and returned to baseline at the end of the session. An interaction between sex, condition, and time was also revealed for ΔHHb (P < 0.05). During the PBC session only, ΔHHb increased in both males and females and remained elevated at the end of the recovery period in males only.

### Cerebral oxygenation during the Stroop tasks

**Figure 6:**
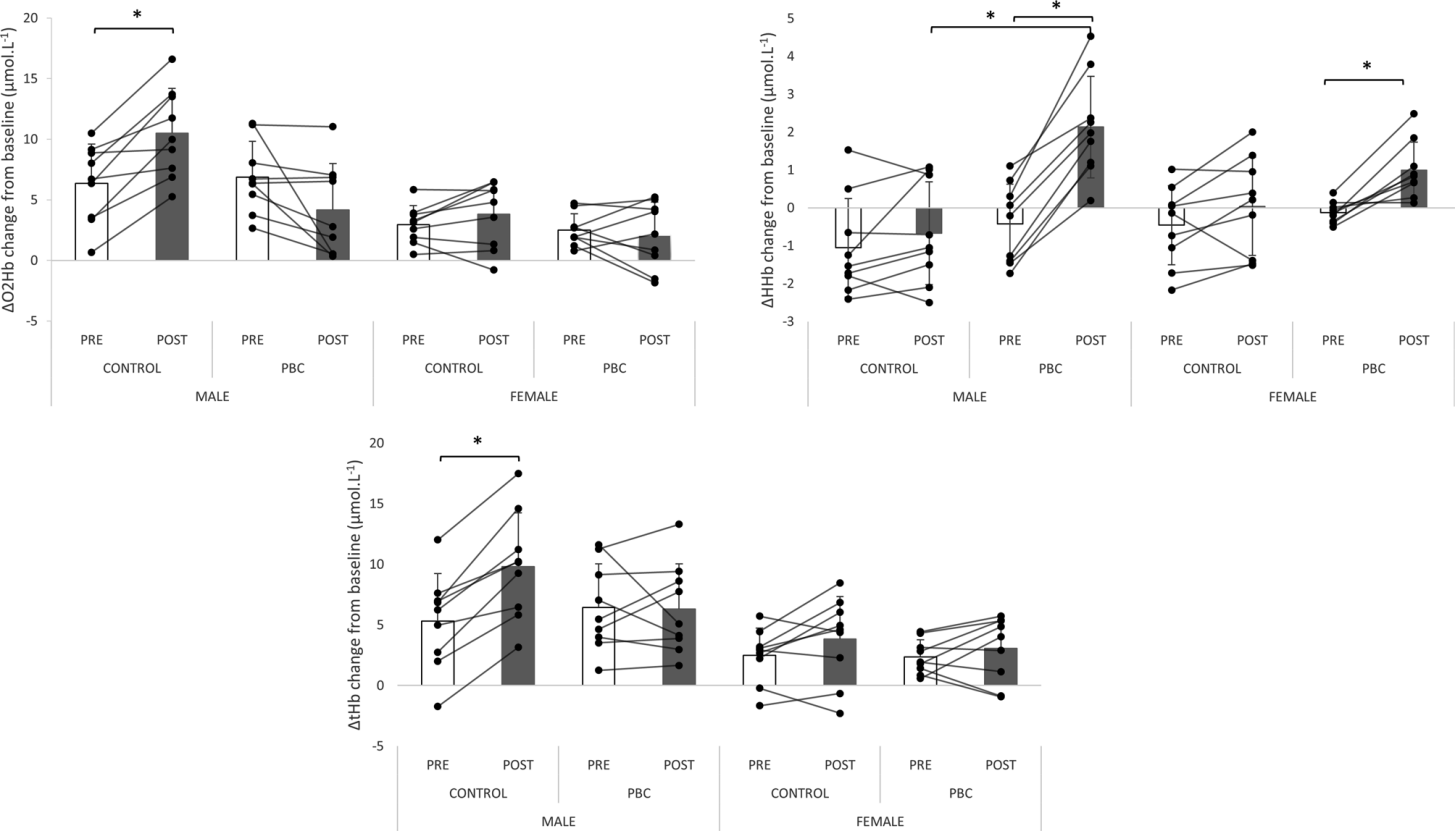
Changes in cerebral oxygenation during the Stroop task performed before and after PBC (partial body cryotherapy) and control conditions, in males and females. *Significant (*P* < 0.05). *O*_*2*_*Hb* oxyhemoglobin, *HHb* deoxyhemoglobin, *tHb* total hemoglobin.

Changes in cerebral oxygenation during the Stroop task (before and after experimental exposition) are presented in Fig. 6. During the Stroop task, regarding ΔO_2_Hb, we found a main effect of Condition (P < 0.05), a main effect of Sex (P < 0.05), a significant interaction Time by Condition (P < 0.05), and Time by Condition by Sex (P < 0.05). During the protocol, we found an increase of ΔO_2_Hb in control condition only in males and not in females. We also found a greater ΔO_2_Hb response for men compared to females. Similarly, ΔtHb increased in males only during the Stroop task. Concerning ΔHHB, we found an increase during the Stroop task in the PBC condition in males and females. Also, we found only in males a difference between ΔHHB in post control conditions and post PBC conditions. After the PBC, ΔHHB was higher than the control condition. This difference was not significant for females.

## Discussion

Using a randomized controlled counterbalanced cross-over design, we tested the effect of PBC on cognitive performance, cardiac autonomic control, and cerebral oxygenation in young healthy males and females. We provide novel data showing that (1) a 3-min PBC exposure improved cognitive inhibition performance assessed in a Stroop test in male participants only, and (2) this was accompanied by a decrease in cerebral oxygenation during exposure (in both males and females) followed by a greater cerebral O_2_ (ΔHHb) extraction in males only, and (3) cardiac autonomic control shifted toward a vagally-mediated tone, in greater proportion in males compared to females. These findings support our hypothesis according to which cold exposure enhances cognitive performance through its actions on the autonomic nervous system and cerebral oxygenation. To our knowledge, this is the first study to report the effect of PBC (a modality of body exposure to extreme cold) on executive function. Concerning cognitive performance, the discrepancies found between males and females might be explained by the greater cardiac autonomic control and cerebral O_2_ extraction (ΔHHb) recorded in males following PBC.

We purposefully selected a PBC system where the head was not exposed to cold, so that NIRS recordings could be conducted during the entire duration of the protocol, including during the session, thus providing the first ever data on cerebral oxygenation during a PBC session. These data could then be linked with HRV and cerebral oxygenation responses to try to understand the mechanisms by which PBC could alter cognitive function. Although this is the first attempt to examine the effects of PBC on cognitive function, comparisons with previous studies using cold exposure can be made. Body exposure to cold was typically shown to impair cognitive performance^32,33,35,47,48^. Besides, previous studies demonstrated that the impairments of cognitive functions by cold exposure follow a dose–response relationship, where the higher the decrease in core temperature the higher the impact on cognitive performance^49^. On the contrary, some studies using very short duration cold exposure reported positive effects on concentration^36^ or cognitive performance^37^. Although the comparison with our study is difficult, Okura et Rikimaru^38^ also recently reported an improvement in executive function in a Stroop task by exposing only the cheeks to cold. In our study, the improvement in cognitive inhibition performance in the Stroop task may be related to a better state of concentration following the PBC session. In our study, we failed to find a positive effect on arousal or perceptual difficulty of the cognitive task. However, the cognitive enhancement could be explained by an increased parasympathetic nervous activity and/or an increased cerebral oxygenation.

In the present study, PBC increased parasympathetic activation in both male and female participants as indicated through higher values of HRV indices of parasympathetic activity (HF, RMSSD, and SD1) and lower values of HR. Based on the neurovisceral model, the increased parasympathetic cardiac tone following PBC could explain the enhanced cognitive performance recorded in the Stroop task. This result is in line with hypotheses according to which higher HRV values would be associated with better performances in a self-regulatory task involving the activation of the prefrontal cortex^20^. More particularly, our results are in accordance with previous studies which reported that the stimulation of vagal nerves (i.e., parasympathetic cardiac tone) modifies cerebral activity and improves cognitive performance^50,51^. Since LF nu might be influenced both by sympathetic and parasympathetic activation^52^, the reduction of LF nu that occurred after PBC might be influenced by a reduced sympathetic activity. Current results corroborate those of previous studies examining the effect of PBC on HRV. A study conducted with forty healthy males, comparing the effect of PBC and WBC on HRV, revealed a significant increase in HF and RMSSD after a single 3-min exposure to both cryostimulation conditions^12^. More recently, a study conducted with thirty healthy males also revealed a significant increase in HF and RMSSD after a single 3-min PBC exposure^53^. These results were confirmed in another recent study by our group and showing that the magnitude of the autonomic response was proportional to the cold stimulus, with the lowest WBC temperature inducing the highest parasympathetic response^13^. Using WBC, Schaal et al.^54^ also reported a significant increase in RMSSD and SD1, concomitant with a decrease in HR following a single 3-min WBC exposure in female athletes. Another point to consider in the present study is that, following the PBC session, the magnitude of the parasympathetic activation was greater in male than female participants, which could explain the between-group differences in the Stroop task. This discrepancy in the HRV responses to PBC might be explained by sexual dimorphism as seen in the present study through differences in body fat percentage. It has previously been hypothesized that women may have a higher insulative capacity compared to men^55^. Women tend to have a higher body fat percentage^56^ which could lead to a reduction of heat transfer toward the periphery of the body^57^. The autonomic response to cryostimulation is likely due to peripheral heat loss, which triggers peripheral vasoconstriction through sympathetic tuning^58^. Consequently, blood volume is redistributed toward the core of the body, leading to increased central blood pressure^59^ causing a modification of the cardiac autonomic balance toward a vagal stimulation^60^. We can, therefore, hypothesize that for the same duration and temperature, women with higher body fat percentage than men, might present a reduced autonomic response to cryostimulation.

As expected, cerebral oxygenation (ΔO_2_Hb) was increased during the cognitive task, associated with a decrease in ΔHHb. This pattern was recorded prior to each exposure (control and PBC) with one recurrent difference that was a greater oxygenation for males compared to females. This difference has already been reported and is explained by a greater amount of hemoglobin in men. Interestingly, after PBC exposure, ΔO_2_Hb and HHB responses recorded during the Stroop task were modified. Indeed, it appeared more markedly in men than in women that the usual lower oxygen extraction under normal conditions is greater after exposure to cold. This change in cerebral oxygenation during the task could be explained by physiological responses that took place during the PBC session. Indeed, during the PBC session, ΔO_2_Hb decreased while ΔHHb increased. Cold exposure, regardless of the cryostimulation technique, is well known to induce a decrease in muscle, limbic, and skin blood flow, due to vasoconstriction^61–64^. However, this phenomenon is still poorly understood at the brain level. Similar to the results of our study, Minnett et al.^65^ reported an increase in ΔHHb associated with a decrease in ΔO_2_Hb during cold-water immersion. The decrease in ΔO_2_Hb and ΔtHb is the result of systemic vasoconstriction from one to stimulation of skin receptors and under the influence of the sympathetic nervous system. To compensate for this phenomenon and avoid syncope, the brain protects itself and ensures that a sufficient level of oxygen is supplied to maintain normal functioning via an increased ΔHHb. Following the PBC session, all values of cerebral oxygenation values went back to pre-exposure level in females, whereas ΔHHb remained higher during the recovery period and until the start of the second Stroop task in males. Such greater cerebral O_2_ extraction recorded in males may explain the improved cognitive performance recorded in the Stroop task performed post-PBC in males compared to females.

Sex-specific perceptual, cognitive, and physiological responses observed in our study find explanation in several previous studies. First, we observed that the thermal sensations between men and women were identical. This result had already been reported^66^. Also, we noticed that the cardiac autonomic nervous responses as well as the cerebral oxygenation responses were less marked in women than in men. Indeed, these results are explained in the results of Solianik et al.^66^, which had shown that for the same dose of cryostimulation (same duration and temperature), men had much greater neuroendocrine responses than that of women. All of this could explain the lack of results on cognitive performance in women in our study. These innovative results open up new perspectives concerning the optimization of exposure to cryostimulation in women. Future research should be encouraged and seek to identify the optimal dose to benefist the most from the effects of cryostimulation in women.

Although this study presents original data and is the first investigating the effects of PBC on cognitive performance and cerebral oxygenation in males and females, its results must be interpreted in light of their limitations. As detailed in the methods section, our population, albeit moderate in size (nine males and nine females), was carefully selected according to strict inclusion criteria, thus providing a homogeneous experimental sample in both groups. However, this moderate sample size can be seen as a limitation and some caution should be taken before generalizing the results of this study.

## Conclusion

In summary, we provide novel data by demonstrating that a single PBC session enhanced cognitive inhibition performance in a Stroop task in males, but not in females. This discrepant result might be explained by a greater parasympathetic cardiac control and cerebral oxygenation following cryostimulation in males compared to females. Taken together, these data suggest selective effects of PBC on the cognitive function of males and females, which opens new research perspectives. Based on the current data, the influence of body composition on the sensitivity to cold should be investigated further.
